# Genetic Diversity among Four Populations of *Aedes aegypti* (Diptera: Culicidae) from Honduras as Revealed by Mitochondrial DNA Cytochrome Oxidase I

**DOI:** 10.3390/pathogens11060620

**Published:** 2022-05-26

**Authors:** Denis Escobar, Bryan Ortiz, Oscar Urrutia, Gustavo Fontecha

**Affiliations:** 1 Microbiology Research Institute, Universidad Nacional Autónoma de Honduras, Tegucigalpa 11101, Honduras; denis.escobar@unah.edu.hn (D.E.); bryan.ortiz@unah.edu.hn (B.O.); 2 Unidad de vigilancia de la salud, Secretaría de Salud de Honduras, Tegucigalpa 11101, Honduras; osurrutia@yahoo.com

**Keywords:** *Aedes aegypti*, genetic diversity, COI, ITS2 rDNA, Honduras

## Abstract

*Aedes aegypti* is a hematophagous and highly anthropophilic mosquito with a wide distribution, particularly in tropical and subtropical regions of the world. *Ae. aegypti* is the main vector of several febrile diseases called arboviruses (dengue, yellow fever, chikungunya, and zika viruses), which represent an important public health problem. Populations of this mosquito were nearly eliminated from the Americas in the mid-20th century; however, after the abandonment of control measures, mosquito populations have been recovering territory, have expanded by anthropogenic mechanisms, and have been joined by new populations reintroduced from other continents. The objective of this pilot study was to determine the genetic variability of *Aedes aegypti* collected in four cities located along the so-called logistics corridor of Honduras, which connects the Caribbean Sea to the Pacific Ocean. We studied the sequences of two molecular markers: the cytochrome c oxidase 1 (COI mtDNA) gene and the internal transcribed spacer 2 (ITS2 rDNA) of 40 mosquitoes. Phylogenetic analyzes show two separate clades with a low number of nucleotide differences per site, three haplotypes, and low haplotype diversity. These results suggest a low genetic diversity in the populations of *Ae. aegypti* in Honduras in relation to that reported in other countries of the Central American isthmus.

## 1. Introduction

*Aedes (Stegomyia) aegypti* (Diptera: Culicidae) is a highly anthropophilic hematophagous mosquito native to Africa. It is considered a highly successful invasive species first introduced to the Americas in the fifteenth century [1]. The mosquito was reintroduced in the 1960s and 1970s after being eliminated from many American countries due to a Pan-American plan based on massive spraying of insecticides [2]. *Aedes aegypti* is the main vector for several viruses, known as arthropod-borne viruses (arboviruses), such as dengue (DENV), yellow fever (YFV), chikungunya (CHIKV), and Zika (ZIKV). The reinfestation of the mosquito in the Americas has resulted in the appearance of several outbreaks of arboviruses during the last five decades and threatens the appearance of new potential arboviruses [3,4].

In Central America, DENV serotype 1 was reported between 1977 and 1978 and was responsible for a generalized dengue epidemic. In 1981, the first cases of hemorrhagic symptoms caused by DENV-2 were reported and were responsible for hundreds of thousands of cases and thousands of deaths [2,5]. From there, the four DENV serotypes circulate endemically in the isthmus. In 2021, the Pan American Health Organization (PAHO) reported 19,753 cases of dengue (and 1,041 severe cases) in Honduras, which represents 26.5% of the total cases in Central America [6]. At the end of 2013, the chikungunya virus (CHIKV) made its appearance in the Caribbean islands. CHIKV is responsible for a less severe disease, characterized by fever and severe arthralgia, which can persist for weeks, months, or years after the acute phase of the disease [7]. Two years later, in November 2015, an epidemic outbreak of a rare neurological disease in newborns caused by ZIKV was recognized in Brazil [8]. Since then, CHIKV and ZIKV have spread rapidly throughout the continent due to wide distribution and the high population density of *Ae. aegypti* in the tropics and subtropics of the Americas, particularly in urban areas that concentrate neighborhoods with characteristics that facilitate the reproduction of the vector, such as the need to store water due to the absence of continuous drinking water service.

The natural history and genetic structure of *Ae. aegypti* in the Americas after its reintroduction half a century ago has been studied on a regional and national scale [9,10,11,12,13,14,15,16,17,18,19,20,21,22,23]. Maternally inherited mitochondrial markers [19,20,23] and, more recently, evolutionarily neutral nuclear markers such as microsatellites or SNPs [9,16] have been used in order to unravel the phylogenetic relationships of *Ae. aegypti*. Many authors agree that there are two main lineages of *Ae. aegypti* circulating in the world, whose most likely evolutionary origins are East and West Africa, respectively [20,24,25,26,27]. This hypothesis is based on a significative genetic differentiation between both populations of mosquitoes in Africa and outside Africa [28,29].

Population structure has been demonstrated in mosquitoes from several countries [30,31,32], while genetic homogeneity and gene flow have been described in other populations [10,33,34,35]. The degree of genetic structure and variability could depend on the success of national vector control campaigns, unplanned urbanization, migratory movements, the flow of international trade from each country, how rapidly mosquito populations expand, the number of colonization events, and the size of the founder populations. Two studies have recently been published that analyze the genetic diversity of *Ae. aegypti* in Central America (El Salvador and Panama), which have provided clues to better understanding the origin, distribution, and structure of the mosquito populations [20,23]. However, there is still a lack of information on the genetic diversity of *Ae. aegypti* in other countries of the Central American isthmus, including Honduras. Consequently, here, we present the results of a pilot study that analyzes for the first time the genetic diversity of *Ae. aegypti* collected in four cities in the country. 

## 2. Results

### 2.1. Sequence Diversity of COI and ITS2

A partial fragment of the COI gene from 40 mosquitoes collected in four cities of Honduras (Choluteca, San Pedro Sula, Tegucigalpa, and Comayagua) was sequenced. Thirty-six high-quality sequences were obtained; however, one of the sequences (code: Choluteca231) was identified as *Aedes albopictus*. No nuclear mitochondrial pseudogenes (NUMT) were found. COI sequences were deposited in GenBank under the accession numbers ON100786–ON100813 and ON100815–ON100816. The sequence of *Ae. albopictus* was deposited under accession number ON117566. In addition, the ITS2 rDNA region of the same 40 mosquitoes was sequenced, but only 15 high-quality sequences were obtained. *Aedes aegypti* ITS2 rDNA sequences were deposited under accession numbers ON118407–ON118418 and ON118554 for *Ae. albopictus*. As far as we know, this is the first report of COI and ITS2 rDNA sequences for both species collected in Honduras.

Genetic variability among the 35 COI gene sequences was assessed, revealing 493 of 505 (97.6%) identical sites, pairwise % identity = 99.7%, nucleotide diversity (π) = 0.002819, and Tajima’s D= −1.4388 (Table 1). On the other hand, sequences from the ITS2 rDNA region showed 356 of 359 (99.2%) identical sites, 99.9% pairwise identity, π = 0.000826, and Tajima’s D = −2.098451. The overall mean distance was 0.00 (S.E. = 0.00) for both analyses. The results of the genetic diversity analysis between COI sequences downloaded from GenBank from mosquitoes collected in other countries, regionally and globally, are shown in Table 1.

### 2.2. Phylogenetic Analysis Based on COI

**Figure 1:**
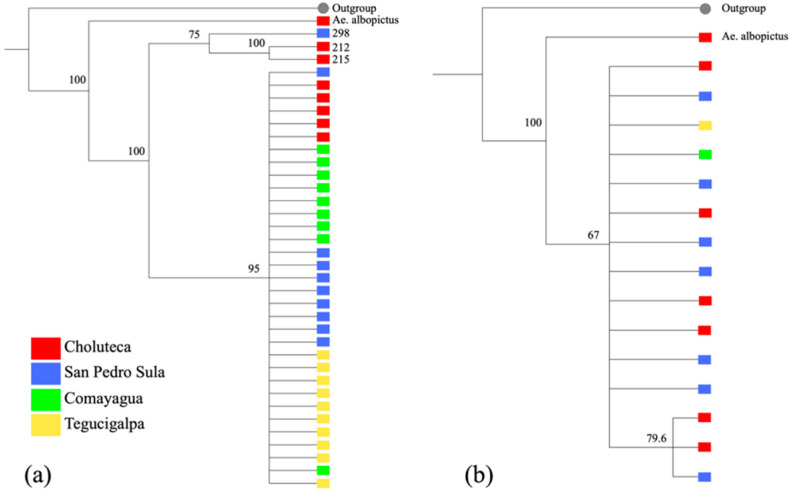
Phylogenetic cladogram of partial sequences of the *Aedes aegypti* (**a**) COI gene, and (**b**) ITS2 ribosomal region, constructed using the Neighbor-Joining method with a bootstrap of 500 replicates. Each color represents a city where the mosquitoes were collected.

Two phylogenetic analyses were performed with the COI and ITS rDNA sequences obtained in this study (Figure 1). The cladogram based on the COI gene showed two well-separated clades with a bootstrap index of 100. The largest clade included 33 individuals collected in the four cities, while a second clade included only three individuals: two from Choluteca and one from San Pedro Sula. There was no separation of individuals based on geography (Figure 1a). The cladogram constructed with 15 ITS2 rDNA sequences also showed two clades but with a smaller bootstrap, and no separation based on geographical distance was evident (Figure 1b).

**Figure 2:**
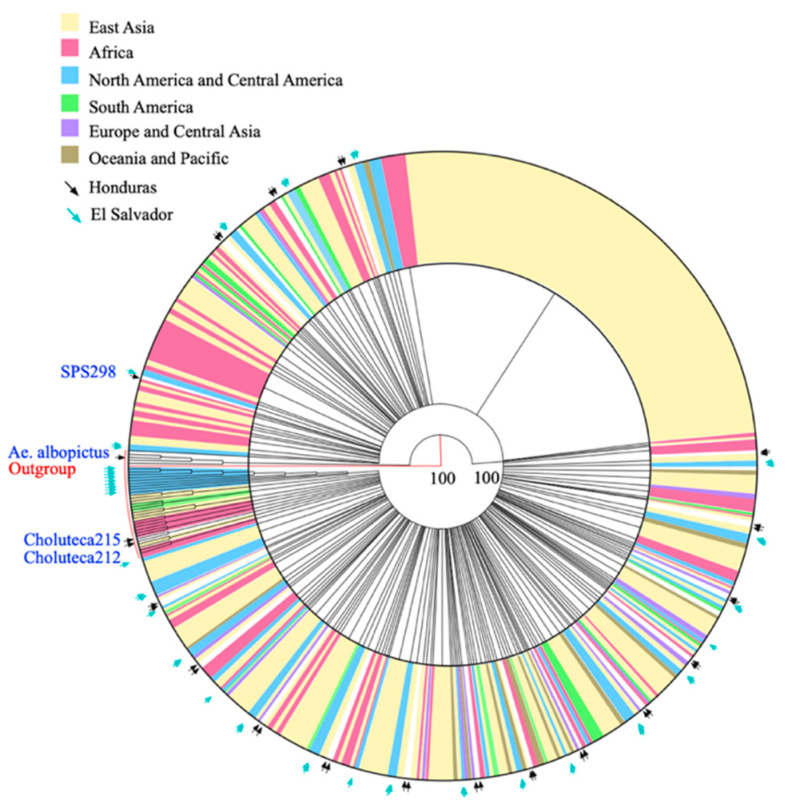
Cladogram (circular tree layout) of partial sequences of the COI gene of *Aedes aegypti* collected in 48 countries. The Neighbor-Joining method was used with a bootstrap of 500 replicates. Each color represents a world region where the mosquitoes were collected. The black arrows indicate the sequences obtained in this study, and the light blue arrows indicate the sequences reported in El Salvador by Joyce et al., 2018 [20].

In addition, a cladogram was constructed with a 450 bp fragment of the COI gene that included 35 sequences obtained in this study and 635 homologous sequences from mosquitoes collected in 48 countries (Figure 2). The tree revealed a clade containing 605 sequences and a smaller clade containing 30 sequences from the following geographic origins: Indonesia (3), Colombia (3), Myanmar (1), Pakistan (1), Bangladesh (1), Kenya (6), India (2), Democratic Republic of Congo (2), El Salvador (9), and Honduras/Choluteca (2). According to this result, there does not seem to be a clear geographical separation for *Ae. aegypti*.

### 2.3. Mitochondrial COI Haplotype Analysis

**Figure 3:**
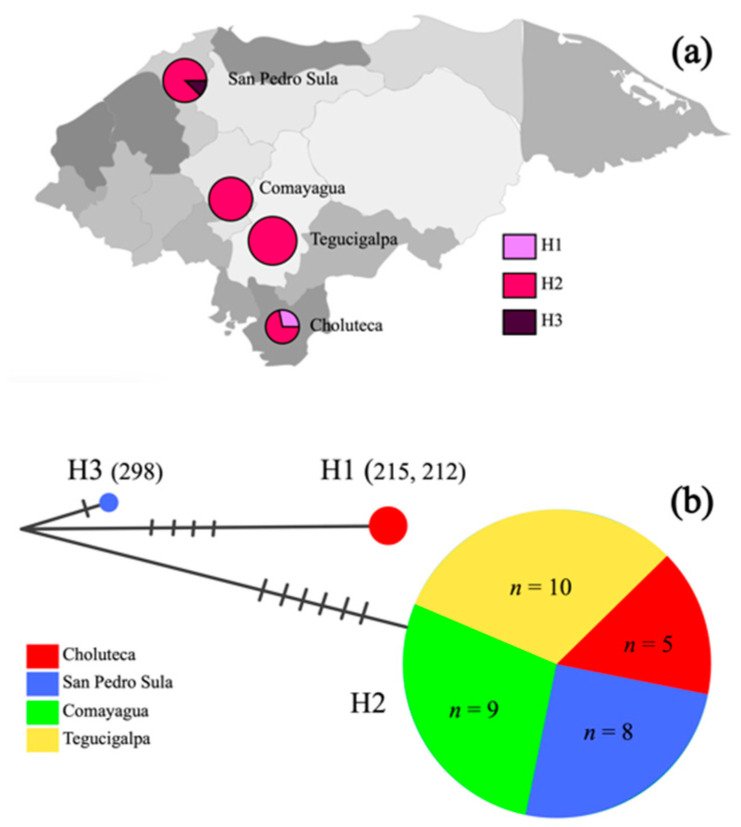
(**a**) Map of Honduras showing three mitochondrial haplotypes (H1–H3) of *Ae. qegypti* by city. The size of the chart is proportional to the number of individuals per haplotype; (**b**) haplotype network based on 35 mitochondrial DNA COI sequences of *Ae*. *qegypti* collected in four cities of Honduras. Perpendicular bars indicate the number of nucleotide polymorphisms between haplotypes.

Three distinct haplotypes were identified out of 35 mosquitoes collected in four cities (Figure 3). Haplotype 1 (H1) included two mosquitoes collected in Choluteca (specimens 215 and 212), the second haplotype (H2) was the most common with 32 individuals from the four cities, and haplotype 3 (H3) included only one individual collected in San Pedro Sula (specimen 298). The haplotype diversity (Hd) of the entire population was 0.1647.

### 2.4. Amino Acid Sequence Analysis

**Figure 4:**
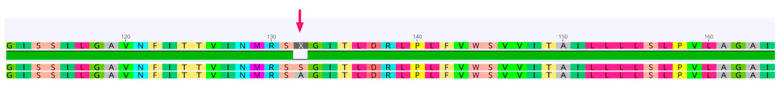
Amino acid alignment of a partial sequence of Cytochrome oxidase 1 protein from *Aedes aegypti* (top) and *Ae. albopictus* (bottom). The red arrow shows the only position where there is a substitution (serine for alanine).

A unique sequence of 168 amino acids was obtained by translating the 35 sequences of the *Ae. aegypti* COI gene. There was no inter-individual variation despite the eight polymorphic nucleotides. The partial sequence of the COI protein was “HPGMFIGNDQIYNVIVTAHAFIMIFFMVMPIMIGGFGNWLVPLMLGAPDMAFPRMNNMSFWMLPPSLTLLLSSSMVENGAGTGWTVYPPLSSGTAHAGASVDLAIFSLHLAGISSILGAVNFITTVINMRSSGITLDRLPLFVWSVVITAILLLLSLPVLAGAITMLL”. The number of identical sites was 167 (99.4%), pairwise % identity was 99.97%, and pairwise % positive (BLSM62) was 100%. The homologous sequence of *Ae. albopictus* showed a single residue difference (serine to alanine) with respect to the protein of *Ae. aegypti* as shown in Figure 4.

## 3. Discussion

This study was a pilot aimed at genetically characterizing *Ae. aegypti* mosquitoes collected in Honduras. Mosquito collections were carried out in four cities located along the so-called logistics corridor of Honduras, which crosses from the Caribbean Sea to the Pacific Ocean. A second objective of the study was to compare the phylogeny of mosquitoes from Honduras with respect to mosquitoes collected in countries on four continents. Our results suggest low genetic diversity in the populations of *Ae. aegypti* from Honduras, based on a low number of nucleotide differences per site (π = 0.0028, and 99.7% pairwise identity), low number of haplotypes (h = 3), and low haplotype diversity (Hd = 0.16). Large-scale studies have shown that populations of *Ae. aegypti* differ in genetic diversity according to geographic region [22]. Due to the high allelic richness of mosquito populations in Africa, it is widely accepted that this continent was the region of origin of *Ae. aegypti* [29]. Less genetic variability outside of Africa suggests that the Americas, Asia, and the Pacific Islands were later colonized by the mosquito [29]. The first massive dispersal out of Africa took place with the transatlantic migratory movements from the Old World to the Americas beginning in the fifteenth century [36], introducing arboviruses such as yellow fever and dengue, which shaped the demography of the Americas during the centuries following the conquest [2,37].

An extensive plan directed by PAHO between 1947 and 1970 aimed to eradicate *Ae. aegypti* from the American continent with the main objective of combating urban yellow fever through the use of insecticides such as DDT [2]. Despite enormous regional efforts, during the second half of 1960, the mosquito was reintroduced to territories where it had previously been eradicated [12], such as the Central American isthmus [20,23]. This led to a bottleneck effect on mosquito populations [9], with the consequent decrease in its genetic diversity. Two studies evaluating the genetic diversity of *Ae. aegypti* in Central America have been published recently. Joyce et al., 2018 sequenced a partial region of the COI gene from 84 mosquitoes collected in six geographic regions of El Salvador [20]. The overall nucleotide diversity was 0.015, with 10 haplotypes and a haplotype diversity equal to 0.61. A second study carried out in Panama analyzed 122 mosquitoes collected in 10 provinces, estimating a nucleotide diversity of 0.0096, 13 haplotypes, and Hd = 0.766 [23].

Both reports suggest a moderately higher genetic diversity in mosquito populations in El Salvador and Panama than that found in Honduras. This difference could be the result of the low number of sequences analyzed in this pilot study (*n* = 36), and another reason behind the low diversity could be that some of the mosquitoes might have been siblings due to the egg collection strategy. In the El Salvador study, a few larvae were collected from each water reservoir [20], and in Panama, only one specimen (larvae or adult mosquito) was collected from each house [23], minimizing the chance of collecting siblings. Furthermore, an entomological collection strategy focused on only four cities and only one neighborhood in each city is also a possible reason behind our results. These are limitations that will have to be overcome in the near future. However, these limitations could explain the low intrapopulation diversity but not the low diversity among the four cities in which the mosquitoes were collected.

Another hypothesis behind the difference between our results and results from neighboring countries could be that mosquitoes from El Salvador and Panama were collected from both urban and rural areas, while mosquitoes from Honduras were collected only from highly urbanized cities, with high connectivity between them. A study based on distribution models of *Aedes albopictus* in Panama showed that road networks alone better explain the geographic expansion of the mosquito [38]; therefore, the logistics corridor linking the four cities where the Honduran mosquitoes were collected could have favored the homogenization of the populations by passive anthropogenic movement [39]. Moreover, the greater flow of international trade observed in Panama and El Salvador could be responsible for multiple reintroductions of mosquitoes from other geographic regions increasing their genetic diversity [23].

Studies based on different nuclear or mitochondrial molecular markers applied to mosquitoes collected in the Americas [9,10,11,16,18,40] report lower genetic diversity than in some Asian countries [25,26,30,41,42,43,44] and most of Africa [22,31,45]. However, regardless of their country of origin, the vast majority of studies worldwide suggest the existence of two distinct lineages of *Ae. aegypti* [11,12,16,19,20,24,26,27,30,46,47]. Our results are consistent with the literature. Despite the small sample size, it was possible to detect two strongly supported phylogenetic clades: a dominant clade made up of 33 individuals and a second minority clade made up of the remaining three individuals. This pattern coincides with that reported by Joyce et al., 2018 in El Salvador and is consistent with the theory of multiple reinfestations in the American continent after the 1970s from African populations or the dispersal of mosquitoes that survived the eradication campaign, as suggested by Eskildsen et al., 2018 [23]. A single dominant haplotype in the four cities of Honduras would indicate a single panmictic population. This could indicate that neither the ecological differences nor the distance that separates the four cities would have any significant effect on the configuration of the mosquito populations. On the other hand, a negative Tajima’s D result could be evidence of a recent expansion after the bottleneck suffered fifty years ago. However, it will be necessary to further expand the number of mosquitoes analyzed, the geographical coverage of the country, and the application of neutral markers such as microsatellites to delve into the population structure of *Ae. aegypti* in the country.

Finally, this study reports for the first time in the country the molecular confirmation of *Ae. albopictus*. Although this species is present on all continents, occupying the same ecological niches as *Ae. aegypti* in the Americas [48], neither do the country’s health authorities carry out routine entomological surveillance nor is its relevance as an arbovirus vector known. Future research could contribute to delving into the population dynamics of *Ae. albopictus* in Honduras.

## 4. Materials and Methods

### 4.1. Study Sites, Mosquito Collection, and Morphological Identification

**Figure 5:**
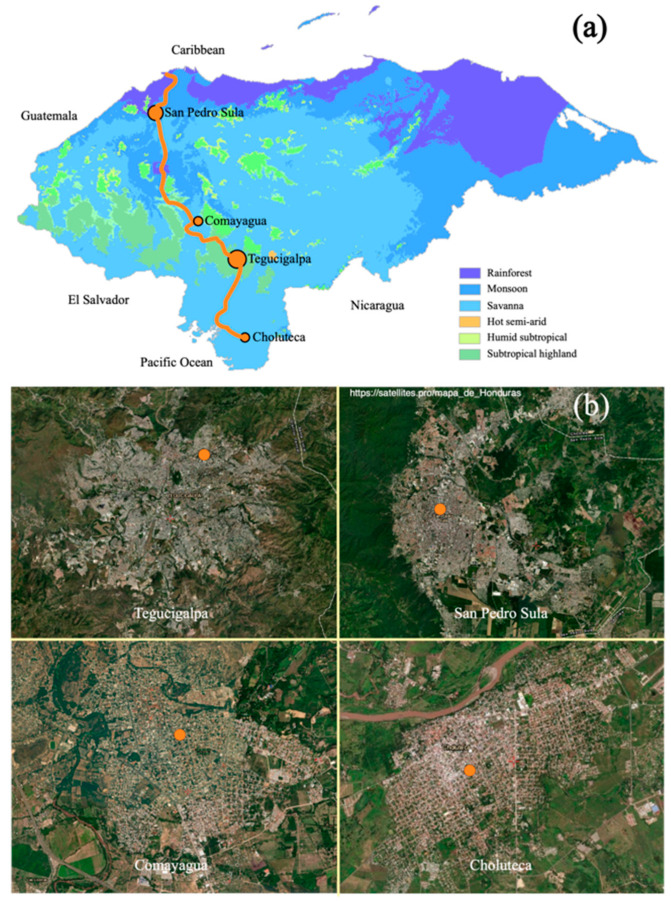
(**a**) Map of Honduras showing the location of four cities where the mosquitoes were collected. The orange line represents the road CA-5 connecting the cities. The map shows the country’s ecoregions according to the Köppen–Geiger climate classification. Adapted from [49]; (**b**) satellite images of the four cities indicating the geographic point (orange dot) where the eggs of the mosquitoes were collected. Adapted from Google.com/maps (accessed on 4 May 2022).

The study sites selected are used as sampling points in routine entomological surveillance for *Ae. aegypti* carried out by the Honduran Ministry of Health. Mosquitoes were collected in four cities with a population greater than 150,000: Comayagua (coordinates: 14.455532, −87.636718), Choluteca (13.2979940, −87.189693), Tegucigalpa (14.1080110, −87.188450), and San Pedro Sula (15.5081440, −88.027036) (Figure 5). The four cities are connected by the country’s main road, called “the logistics corridor” or CA-5, which connects the Caribbean Sea with the Pacific Ocean, and through which most of the movement of goods and people in the country takes place. Despite being located in a relatively small country (112,492 km^2^), the ecological conditions of the four cities are different. San Pedro Sula is located in a valley 83 meters above sea level with Atlantic influence that receives a lot of rain and is bordered by the Merendón mountain range, considered a tropical forest according to the Köppen–Geiger climate classification [49]. Comayagua is located in a valley in the central region of the country, with a savannah-type ecosystem at 594 m.a.s.l. Tegucigalpa, the capital city, rises 990 m.a.s.l. and is surrounded by mountains with subtropical coniferous forest, while Choluteca is located near the coast of the Gulf of Fonseca, with a drier climate at 65 m.a.s.l.

A sentinel site was selected in each city in coordination with the Ministry of Health. *Aedes aegypti* eggs were collected between January and March 2018 using ovitraps. Briefly, ovitraps were prepared using 10% hay infusion over paper towel as the substrate for oviposition [50]. The ovitraps were distributed in five to ten houses at each site with a distance of 200 meters between them and were visited weekly until eggs were found. Egg-bearing papers with more than 100 eggs were transported to local insectaries in each city where they were hatched and raised to the adult stage. Ovitraps with less than 100 eggs were discarded assuming that a single adult mosquito had laid eggs. In each city, all the larvae were grouped together and then reared to adults.

Standard rearing conditions were used (26–29 °C, with 70–90% relative humidity, and a 12:12 photoperiod) [51]. Adult mosquitoes were confirmed to be *Ae. aegypti* using morphological characteristics described in taxonomical keys [52]. All female mosquitoes were stored at −20 °C until subsequent molecular analysis in Tegucigalpa.

### 4.2. DNA Extraction, Gene Amplification, and Sequencing

A subset of ten mosquitoes per site was randomly selected for DNA extraction. DNA was extracted from each specimen following the ReliaPrep™ Blood gDNA Miniprep System (Promega, Madison, WI, USA) protocol. Following the manufacturer’s instructions, mosquito maceration was carried out with a pestle in a 1.5 mL conical tube. Lysis was performed at 56 °C for one hour. DNA was eluted in 80 μL of elution buffer and stored at −20 °C until further use.

Two molecular markers were amplified: the cytochrome c oxidase 1 gene (COI) and the internal transcribed spacer 2 (ITS2 rDNA). COI was amplified with the following primers: LCO1490 (5′-GGT CAA CAA ATC ATA AAG ATA TTG G-3′) and HCO2198 (5′-TAA ACT TCA GGG TGA CCA AAA ATC A-3′) [53]. PCR reactions were carried out in a volume of 50 μL, with 25 μL of Taq Master Mix 2× (Promega, Madison, WI, USA), 2.0 μL of each primer (10 μM), 2 μL of acetylated bovine albumin (BSA) (10 mg/mL), 4 μL of DNA, and nuclease-free water. The PCR program was as follows: 1 cycle at 95 °C for 10 min, 37 cycles at 94 °C for 1 min, 48 °C for 1 min, 72 °C for 1 min, and 1 cycle at 72 °C for 7 min. PCR products were separated by electrophoresis in 1% agarose gels with ethidium bromide.

For ITS2 amplification, PCR reactions were performed using the universal primers 5.8S (5′-ATC ACT CGG CTC GTG GAT CG-3′) and 28S (5′-ATG CTT AAA TTT AGG GGG TAG TC-3′) [54]. Reagent concentrations were as follows: 25 μL of Taq Master Mix 2×, 2 μL of each primer 10 μM, 2 μL of DNA, and water for a total reaction volume of 50 μL. PCR amplifications were performed with the following conditions: 94 °C for 2 min, 34 cycles of 94 °C for 30 s, 57 °C for 30 s, 72 °C for 30 s, and a final extension step at 72 °C for 10 min.

The amplification products of both loci (COI and ITS2 rDNA) were sequenced on both strands using the same primers for the PCR. Sequencing services were provided by Psomagen^®^. The sequences were edited with the Geneious^®^ 9.1.7 software and deposited into the NCBI GenBank. All sequences were submitted as queries to NCBI through the BLAST tool [55] under default parameters to identify the most similar sequences in the GenBank nucleotide collection.

### 4.3. Data Analysis

Thirty-six partial sequences of the mitochondrial COI gene of *Ae. aegypti* were analyzed. For sequence analysis, only those showing a %HQ index greater than 98.5% after trimming were used. Sequences were aligned with the Geneious algorithm of the Geneious^®^ 9.1.7 software. A homologous sequence from *Anopheles albimanus* was included in the alignment as an outgroup. Sequences with insertions or stop codons that might suggest nuclear mitochondrial pseudogenes were searched for (NUMT) [56]. Phylogenetic analysis used the Tamura–Nei distance model, the Neighbor-Joining method, and a bootstrap of 500. A cladogram (rooted tree layout) was constructed including a homologous sequence of *Ae. albopictus* obtained in this study. The length of the nucleotide sequences, the number and percentage of identical sites, and the pairwise % identity were calculated. MEGA v11.0 software [57,58] with 500 bootstrap replicates was used to calculate the overall mean distance, standard error (S.E.), number of segregating sites (S), nucleotide diversity (π), and Tajima test statistic (D). Sequences of the ITS2 rDNA region were also analyzed, and a cladogram was constructed.

Two sets of analyses similar to those described above were performed using homologous COI sequences from *Ae. aegypti* downloaded from GenBank and obtained from mosquitoes collected in other countries. The first analysis included 669 sequences from 48 countries on five continents. The second analysis included 217 sequences from seven countries in the central region of America (Mexico, Guatemala, El Salvador, Honduras, Costa Rica, Panama, and Colombia). In both analyses, the sequences were edited to keep exactly the same size (450 bp and 352 bp, respectively). A phylogenetic cladogram (circular tree layout) was constructed using 669 homologous sequences (36 obtained in this study and 633 downloaded from Genbank), using the Tamura–Nei distance model, the Neighbor-Joining method, and a bootstrap of 500 replicates with a homologous sequence of *Anopheles albimanus* as an outgroup.

The number of haplotypes (h) and haplotype diversity (Hd) were calculated according to the nucleotide sequences with Dna SP software v. 6.12.03 [59]. Alignment sequences were imported, and parameters were adjusted for mitochondrial DNA with genetic code for *Drosophila* mtDNA. Haplotype data were generated using the Roehl data file function and default parameters.

Nucleotide sequences obtained in this study were translated using the correct open reading frame (ORF) using the invertebrate mitochondrial genetic code in the Geneious^®^ 9.1.7 software. Residue alignment was performed, and the number of identical sites, pairwise % identity, and pairwise % positive (BLSM62) were calculated.

## 5. Conclusions

This is, to our knowledge, the first study carried out in Honduras that analyzes the genetic diversity of *Ae. aegypti*. The results of this pilot seem to indicate the presence of two mosquito lineages and three haplotypes (one dominant haplotype) with low genetic diversity and no apparent population structure, which coincides with what has been reported previously in the Central American region. Future studies that expand the number of mosquitoes and the number of cities will shed more light on the evolutionary history of *Ae. aegypti* in Central America more than half a century after its reintroduction.

## Figures and Tables

**Table 1:** Genetic variability parameters estimated for partial sequences of the COI gene of *Aedes aegypti*.

Statistics	Honduras	Region ^1^	World ^2^
Length (bp)	505	352	450
Number of sequences	35	217	670
Identical sites (%)	493 (97.6%)	288 (81.8%)	323 (71.8%)
Pairwise % Identity	99.7%	98.9%	99.3%
Overall mean distance	0.00	0.01	0.01

^1^ Sequences of mosquitoes collected in Mexico, Guatemala, El Salvador, Honduras, Costa Rica, Panama, and Colombia. ^2^ Sequences of mosquitoes collected in 48 countries on five continents.

## Data Availability

Not applicable.
